# Validity and Reliability of the Hungarian Version of Aberdeen Varicose Vein Questionnaire

**DOI:** 10.3390/ijerph19031639

**Published:** 2022-01-31

**Authors:** Gabriella Kiss, Dorottya Szabó, Eva Tékus, Gábor Jancsó, Endre Arató, Alexandra Makai, Melinda Járomi, Tibor Mintál

**Affiliations:** 1 Sports Medicine Center, Medical School, University of Pécs, Akac Street 1, H-7632 Pécs, Hungary; dorottya.szabo@aok.pte.hu (D.S.); eva.tekus@aok.pte.hu (E.T.); mintal.tibor@pte.hu (T.M.); 2 Department of Vascular Surgery, Clinical Center, University of Pécs, H-7624 Pécs, Hungary; jancso.gabor@pte.hu (G.J.); arato.endre@pte.hu (E.A.); 3 Institute of Physiotherapy and Sport Science, Faculty of Health Sciences, University of Pécs, H-7621 Pécs, Hungary; alexandra.makai@etk.pte.hu (A.M.); jaromi@etk.pte.hu (M.J.)

**Keywords:** venous diseases, varicosity, quality of life, AVVQ, SF-36

## Abstract

Purpose: The aim of our study was to translate the Aberdeen Varicose Vein Questionnaire (AVVQ) into Hungarian, and to investigate the validity and reliability of the Hungarian AVVQ, as well as to assess the health-related quality of life in patients with varicose veins of the leg. Methods: 374 adults participated in this study who were divided into two groups (varicose vein, healthy). We analyzed internal consistency, convergent validity (using the 36-Item Short Form Survey, SF-36), repeatability, and intra-class correlation coefficient of the Hungarian AVVQ. Regarding discriminant validity, we determined the scores of the Hungarian AVVQ in both groups using the Mann-Whitney U-test. Results: The Cronbach-alpha value was 0.890, while the correlation coefficient was R = 1.000. According to the results of the convergent validation, the scores of pain and dysfunction moderately correlated with some scores of the SF-36. The score of cosmetic appearance had a relationship with many scores of the SF-36. We registered a significant relationship between the score of extent of varicosity and some scores of the SF-36. There was significant correlation between the score of complications and numerous scores of the SF-36 (physical functioning, role limitations due to physical health, pain and general health). The score of pain and dysfunction, cosmetic appearance, extent of varicosity, complications and total score of the Hungarian AVVQ showed a significant difference between both groups. Conclusions: The Hungarian AVVQ was a reliable and a valid tool to assess the health-related quality of life among patients with varicose veins and was a useful tool to justify the further treatment of the patients.

## 1. Introduction

Among chronic vein diseases, varicosity is the most common, affecting nearly a third of the European population [1]. Moreover, venous diseases of the lower limbs are a major cause of medical expenditure in the western world [2]. International studies have shown a high prevalence of chronic venous disease (75.2%) [3], with 40% of men and 32% of women suffering from varicose veins. According to Ortega and coworkers [4], the estimated prevalence of chronic venous disease was between 60–80%, and more than 80% of the total population suffered from minor venous disorders [5]. Bihari and coworkers [6] described the prevalence of lower limb varicose veins (57.1%) in Hungary, a value similar to other European countries.

Assessing quality of life, especially disease-specific quality of life and making its changes measurable is an increasingly important patient-oriented approach. A number of health assessment tools and questionnaires have been developed and approved worldwide to more accurately assess health status [7,8]. Few tools are available for estimating the quality of life of patients with venous diseases and these are compared with other diseases. These questionnaires are usually available in English, such as the Chronic Venous Insufficiency Questionnaire [9], or the AVVQ [10]. In addition, a multistep process is required to adapt a questionnaire, which includes the translation, the cultural adaptation, and validation of the recommended instrument [9].

AVVQ was developed and validated by Garratt to measure the quality of life of patients suffering from lower-limb varicose veins [10]. The perceived health status of varicose veins patients, measured by the SF-36, was significantly lower compared to the general population, where the proportion of women was lower [10]. Klem and colleagues (2009) validated the AVVQ questionnaire in the Netherlands and in the Dutch-speaking region of Belgium, where they found a high test (99%) and retest (97%) response [11]. Other research groups validated this questionnaire with SF-36 in Portuguese and found the AVVQ to have high inter- and intra-observer reliability with internal consistency ranging from excellent to moderate for most domains [12]. The test-retest reliability of Turkish AVVQ was between good and excellent (Kappa 0.447-1) and the validity of this questionnaire was found to be good [13]. The reliability and validity of the Persian AVVQ questionnaire were determined by Neamatshahi and colleagues using the Cronbach alpha coefficient of face, content, criterion, and construct validity and reliability [14].

The SF-36 is one of the survey questionnaires with multi-dimensional scales used to investigate the general quality of life. Validation of AVVQ with SF-36 was performed based on Garratt.

No validated questionnaire on the quality of life of varicose vein patients has been published in the Hungarian literature so far [12].

The aim of our study was to translate the AVVQ into Hungarian, and to test the validity and reliability of the Hungarian version of Aberdeen Varicose Vein Questionnaire (AVVQ-H), and to assess the health-related quality of life of patients with lower limb varicose veins using this questionnaire.

Our hypotheses were as follows. First we hypothesized that the AVVQ-H was a valid and reliable tool of assessing the health-related quality of life of patients with venous disease. Second, we hypothesized that the results of the Hungarian-language version of the Aberdeen Varicose Vein Questionnaire would show a significant correlation with the results of the SF-36.

## 2. Materials and Methods

### 2.1. Participants

Our cross-sectional study included 374 adult individuals (of both sexes). They were using simple, non-probability sampling based on the results of previous studies [10,11,12,13,14]. In the survey, the subjects were divided into two groups. The patient group included those with varicose veins. Inclusion criteria were: participants aged 18–65 and voluntary participation. Exclusion criteria were: non-Hungarian native speakers, patients with other pulmonary, cardiological comorbidities, patients with reading- or speech impairment and patients who had a deep vein thrombosis or pulmonary embolism within six months of the study. Those with a BMI over 35 were also excluded [15]. The healthy group included participants with no history of venous diseases in their medical records. Exclusion criteria for the healthy group were non-Hungarians and patients with other severe musculoskeletal, internal, neurological and venous diseases. Those with a BMI over 35 were also excluded. The full dataset presented in this study is openly available in FigShare at https://doi.org/10.6084/m9.figshare.14865129.v1 (accessed on 10 January 2022).

Informed written consent was obtained from all participants in the study. The ethical approval of the research was granted by the Ethics Committee of our university (Permit number: 6922/2017) and were performed in full accordance with the Helsinki Declaration. 

### 2.2. Aberdeen Varicose Vein Questionnaire (AVVQ)

AVVQ (Table 1) was developed and validated by Garratt and colleagues in 1993. The questionnaire assesses health-related quality of life in patients with lower limb venous disease and consists of 13 questions related to varicose veins. Question 1 originally asked respondents to draw schematic front and back views of the lower limbs showing the location of the varicose veins. Due to the online format of the questionnaire, patients usually did not draw anything in question 1 and omitted this diagram. Several of them did not understand and solve this task alone, therefore this question was modified so that respondents had to select from seven regions where they had varicose veins. Questions 2–6 and 12–13 asked about the two week period prior to the survey. Respondents were given more options. They could choose the frequency of their pain, whether they had worn medical socks/stockings on their own initiative/as prescribed by a doctor, whether they had experienced itching and to what extent their varicose vein problems affected their work and leisure activities. Questions 7–11 referred to the usual symptoms (e.g., whether the patients had experienced purple discolouration in the varicose vein area, eczema around the ankle or varicose vein complaints associated with pressure sores), not to symptoms in the last two weeks. The questionnaire also included questions as to whether patients were worried about the appearance of varicose veins and the extent to which the appearance of veins affected their daily dressing. Questions 1–2 and 5–9 asked separately about the right and left side [10]. 

The primary author identified four important health-related subscales, namely pain and dysfunction (including questions: 2, 3, 12, 13), cosmetic appearance (including questions: 10, 11), degree of varicosity (including questions: 1, 5, 7), and complications (including questions: 4, 6, 8, 9). The AVVQ total scale ranged from 0–100, where 0 is the best and 100 is the worst result. The AVVQ questionnaire was scored according to the international guidelines provided by the primary authors [10,16]. Questionnaires were completed using LimeSurvey software. The software was available by email invitation.

### 2.3. 36-Item Short Form Survey (SF-36)

To validate AVVQ we used the SF-36 developed by the Boston Health Research Institute [17]. Participants completed the SF-36 questionnaire measuring their health status, which was validated and published in Hungarian by Czimbalmos and colleagues in 1999 [18].

The SF-36 questionnaire is one of several scales that measures the general quality of life. The Hungarian version of the test has been validated and the normal values of healthy respondents in Hungary are known. It measures a patient’s opinion regarding their own health, condensed into 36 questions. It contains eight sets of questions on quality of life, which are called scales. These are physical activity (PF), role limitations due to physical health (RP), physical pain (BP), general health perception (GH), vitality (VT), social functioning (SF), role limitations due to emotional problems (RE) and general mental health (MH). In the SF-36 assessment process, patients are given scores between 0–100 on each scale according to their responses. A score of 0 represents the worst quality of life, while 100 represents the best quality of life. RF, RP, BP and GH are used to assess physical health, while the RE, VT, MH, and SF assess mental health. The SF-36 questionnaire is used in a number of clinical areas in both medical and physiotherapy research where we want to measure changes in health status following interventions [19,20,21].

### 2.4. Translation and Validation of the Questionnaire with Beaton Six-Step Principle

**Figure 1:**
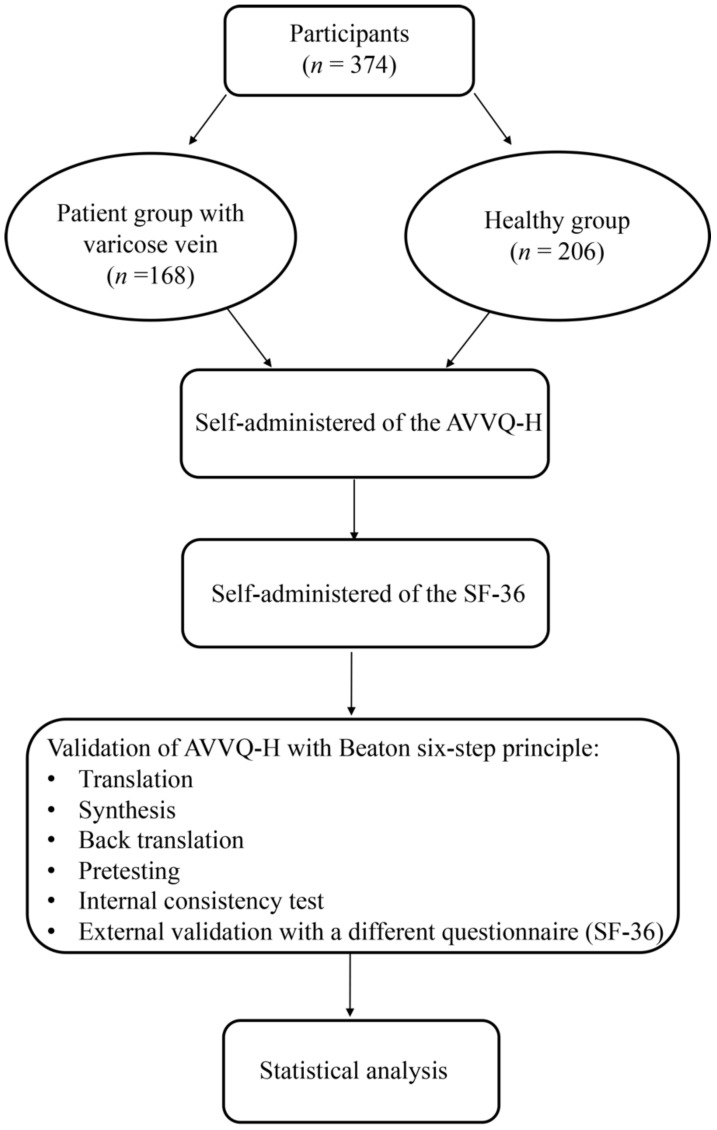
The study protocol. AVVQ-H: Hungarian version of Aberdeen Varicose Vein Questionnaire, SF-36: 36-Item Short Form Survey.

The quality of the validation process was evaluated using COSMIN checklists [22]. The translation and validation of the AVVQ questionnaire in Hungarian followed the six-step principle formulated by Beaton and coworkers in 2000 (Figure 1): translation, synthesis, back translation, pretesting, internal consistency test, and external validation with a different questionnaire. The first step in the adaptation process is always the first translation. The objective suggestion is that at least two translators should translate from the original language into the target language. In the second step, these two translators and an observer recording the process synthesize the results of the translations. After the synthesis, the third step is for the translator who is not familiar with the original text to translate the questionnaire back into the original language. The purpose of the validity checking process is to check whether the translated version reflects the same content as the original version. This step allows us to effectively identify the questionable items in the translations. In the fourth step, a committee of experts consolidates all the versions of the questionnaire and develops the beta version to test it. Accordingly, the committee will review all translations and draw conclusions on each of the disputed terms. The original translation and all translations will be made available to the committee as well as the written reports. The penultimate stage of the adaptation process is the pretesting, which is carried out with a group of 30 participants. Each participant completes the questionnaire and provides additional feedback on each question and on their answers. The sixth step is the submission of the documentation to the developers or the coordinating committee for their evaluation of the adaptation process. In this step the committee does not change the content of submitted materials [23].

Problematic expressions have been corrected. We replaced “support/medical socks-stockings” with “compression stockings”. Question 1 of the questionnaire contains a drawing of the lower limbs from the front- and back with the location of the varicose veins drawn. Requesting a drawing from Hungarian patients in a questionnaire sounded unfamiliar. As a result, drawings were often omitted. Therefore, respondents’ attention had to be directed to the drawing several times so they were included in the questionnaire with special emphasis.

Following the discussion with the authors, and upon their recommendation, the SF-36 questionnaire was chosen for convergent validation of the AVVQ-H. Since then no other validated questionnaire measuring the quality of life of varicose vein patients has been available in Hungarian. Subsequently, patients provided written consent and completed the AVVQ-H and SF-36 questionnaires.

### 2.5. Statistical Analysis

IBM SPSS 27.0 version was applied for the statistical analysis. Mean and standard deviation of the variables were calculated to present the descriptive characteristics of the sample. The internal consistency of the questionnaire was measured by calculating the Cronbach′s alpha value, which can be considered reliable from the value of 0.7 [9,10]. Correlation of the AVVQ-H with the SF-36 questionnaire was examined with Spearman’s rank correlation coefficient. Repeatability of the survey was carried out on a sample of sixty responders with the test-retest method, and the inter-class correlation coefficient in the course of statistical analysis was again calculated for statistical analysis.

A Mann-Whitney U-test was used to determine discriminant validity and differences between healthy participants and venous patients using AVVQ-H. Our results were considered significant in case of *p* < 0.05.

## 3. Results

### 3.1. The Characteristics of the Sample

In our study there were 168 participants with varicose veins and 206 healthy participants. Of the people with varicose veins, 84% were women and 16% were men, while 76% of the healthy were women and 24% were men. Sample characteristics are summarized in Table 2.

### 3.2. Internal Consistency and Test-Retest Reliability of the Questionnaire

The reliability of the questionnaire was analyzed using Cronbach′s alpha values. The statistical analysis shows that the AVVQ-H is reliable and the internal consistency of the questionnaire is appropriate (Cronbach-alpha = 0.890).

The reliability of the questionnaire was further assessed with the test-retest method and was determined with the correlation coefficient between classes. The AVVQ-H’s test and retest scores were the same, so the correlation coefficient was 1.000. This value demonstrates that the results of the first and second survey were consistent for sixty subjects.

### 3.3. Correlation between the Subscale of AVVQ-H and the Scale of the SF-36

The relationship between the AVVQ-H subscale scores and SF-36’s scale scores were analyzed using Spearman’s rank correlation test (Table 3). According to our correlation coefficients, the scores of the two questionnaires showed a significant correlation with moderate closeness. Two scores of SF-36 domains (emotional well-being, social functioning) did not correlate with any scores of AVVQ-H subscales.

AVVQ-H total scores were significantly correlated with some SF-36 scales (physical functioning, role limitations due to physical health, energy/fatigue, pain, general health).

In the convergent validation test, significant correlation was found between the pain and dysfunction score (subscale of the AVVQ-H) and some domains of SF-36 (physical functioning, role limitations due to physical health, role limitations due to emotional problems, pain, general health). The cosmetic appearance score (a subscale of the AVVQ-H) significantly correlates with SF-36’s physical functioning, role limitations due to physical health, pain and general health. We found significant relationship between the extent of varicosity score (subscale of the AVVQ-H) and scores of the SF-36 scales (physical functioning, role limitations due to physical health, pain, general health). A significant association was found between the varicosity extent score (subscale of the AVVQ-H) and scores of the SF-36 scales (physical functioning, role limitations due to physical health, pain, general health). The energy/fatigue score correlated with two subscales of the AVVQ-H (degree of varicosity, complications).

### 3.4. Discriminant Validity 

**Figure 2:**
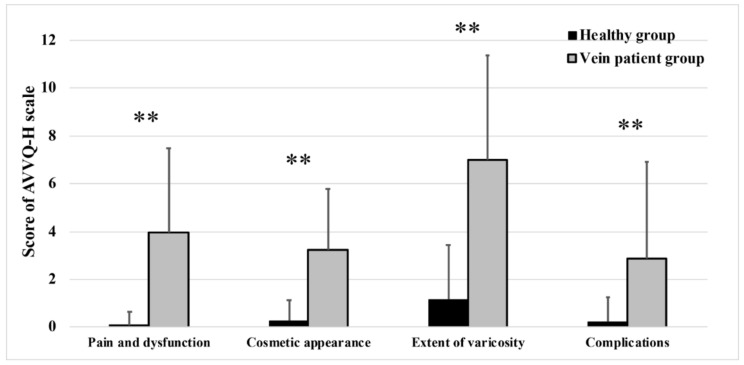
Discriminant validity of the AVVQ-H scores among the healthy and patient groups Mean ± SD, ** Significant difference (*p* < 0.001) between healthy and vein patient group.

An analysis of discriminant validity can measure the differences between the total AVVQ-H score of venous patients and healthy participants, as well as the score of the AVVQ-H subscales. Based on the results of the Mann-Whitney U test, significant differences were found in the total AVVQ-H score (*p* < 0.001) and numerous scores of the AVVQ-H subscales (pain and dysfunction, cosmetic appearance, degree of varicosity, complications) between venous patients and healthy individuals (Figure 2). In all cases, the healthy group scored better in AVVQ-H subscales and on the total score.

## 4. Discussion

### 4.1. Statement of Principal Findings

Our research focused on the validity and reliability of the AVVQ-H, and on the health-related quality of life of patients suffering from lower limb varicose veins using this questionnaire. According to the statistical analysis (internal consistency, test-retest reliability, Spearman’s rank correlation test), the AVVQ-H is a reliable and valid instrument for measuring health-related quality of life. 

### 4.2. Interpretation within the Context of the Wider Literature

For the internal consistency of our survey, Cronbach′s alpha (α = 0.890) was considered appropriate and comparable to that described by Garratt et al. (Cronbach’s α of 0.72) [10], Smith et al. (Cronbach’s α of 0.74) [24] and Klem et al. (Cronbach’s α of 0.76) [11], and Neamatshahi et al. (Cronbach’s α of 0.71) [14].

The reliability of the AVVQ-H test-retest was determined using the inter-class correlation coefficient, which was 1.000 indicating identical results at both test time points. The Hungarian sample showed a higher inter-class correlation coefficient of AVVQ-H than for the Dutch Klem et al. [11] and the UK populations [24]. The similarity of the AVVQ-H results in test and retest can be explained by the fact that the first test was conducted one week later than the second test (retest). In question 1 of the AVVQ-H the Hungarian patients were unfamiliar with the request of drawing their veins so an explanatory sentence was added to the questionnaire and this did not cause any problems afterwards.

The results of the Spearman’s rank correlation test showed that the AVVQ-H subscale and the scale of SF-36 were often correlated with moderate closeness, except for emotional well-being and social functioning (among SF-36 scales). Contrary to that, Leal and colleagues observed [9] correlations between these two SF-36 subscales and some subscales of AVVQ-Brazil. The absence of or different correlations between AVVQ-H subscales and SF-36 scales might have caused SF-36 to be a specific health-related quality of life questionnaire instead of a special mental health or social functioning- related survey [9,25].

Overall, all subscales of AVVQ-H (pain and dysfunction, cosmetic appearance, extent of varicosity, complications) and the total questionnaire score show a significant correlation with the SF-36 dimensions of physical functioning, role limitations due to physical health, pain, and general health. The total score of AVVQ-H correlates with the SF-36 energy/fatigue scale. Previous research [9,10] has described a correlation similar to our study between the varicose vein score (equal with total score of AVVQ-H) and each dimension and scale of the SF-36, suggesting similar reliability with the Hungarian AVVQ.

Furthermore, there is also a relationship between pain and dysfunction (subscale of AVVQ-H) and role limitation due to emotional problems (scale of SF-36), the same as was reported by Leal and coworkers [9]. However, the energy/fatigue scale of SF-36 correlated with two subscales of the AVVQ-H (extent of varicosity, complications), which is different from Leal’s results [9].

A discriminant validity analysis revealed several differences between venous patients and healthy participants in AVVQ-H subscale scores and total score. As expected, the health-related quality of life differs between venous patients and the healthy target group, with the healthy group always showing better scores.

### 4.3. Implications for Policy, Practice and Research

The AVVQ-H can help healthcare professionals to justify surgical treatment of varicose veins and to identify complementary therapies (e.g., physical activity) for patients.

According to our findings, the AVVQ-H was a reliable and a valid tool for assessing health-related quality of life among patients with varicose veins and a useful tool to justify further treatments of the patients. The translated AVVQ has now been validated into Hungarian to measure health-related quality of life of the varicose vein patients. In addition, our results suggested that higher retrained physical function may help to improve the quality of life and reduce physical and mental symptoms in both patients and healthy participants. For comparing the health-related quality of life in patients with varicose veins that are of different nationalities, the AVVQ-H questionnaire, as a validated and translated questionnaire is a suitable tool.

### 4.4. Strengths and Limitations

The main strength of our study was the investigation of the validity and reliability of the AVVQ-H, and the assessment of health-related quality of life among patients suffering from lower limb varicose veins, as quality of life for these patients received little or no attention in Hungary in this regard. Moreover, this questionnaire is a reliable tool for the objective measurement on some of the problems of Hungarian patients with varicose veins in clinical practice and the related quality of life in an international context.

A limitation of this research is that the clinical-etiology-anatomy-pathophysiology (CEAP) classification of patients with varicose veins was not determined, so we could not describe the impact of clinical manifestations of chronic venous disorders on a patient’s quality of life.

## 5. Conclusions

The Hungarian AVVQ was a reliable and a valid tool to assess the health-related quality of life among patients with varicose veins and was a useful tool to justify the further treatment of the patients.

## Figures and Tables

**Table 1:** Characteristics of the AVVQ Questionnaire.

Questions	Content	Health Related Subscales
1	Distribution of veins	Degree of varicosity
2	Duration of pain	Pain and disfunction
3	Duration of analgesia	Pain and disfunction
4	Degree of ankle swelling	Complications
5	Use of support stockings	Degree of varicosity
6	Extent of itching	Complications
7	Any discoloration	Degree of varicosity
8	Rash or eczema	Complications
9	Skin ulcer	Complications
10	Degree of concern at appearance	Cosmetic appearance
11	Influence of choice of clothes	Cosmetic appearance
12	Interference with work, etc.	Pain and disfunction
13	Interference with leisure	Pain and disfunction

**Table 2:** Main characteristics of the sample.

	Healthy Group (*n* = 206)		Patient Group (*n* = 168)	
	Mean	SD	Mean	SD
Age	30.22	14.48	50.96	11.73
BMI	24.48	4.80	27.84	5.73
Years of disease	-	-	7.69	7.79

SD: standard deviation, BMI: Body mass index.

**Table 3:** Spearman’s rank correlation analysis between the AVVQ-H subscales and SF-36 scales.

		PF	RP	RE	EF	EWB	SF	PN	GH
Pain and dysfunction	R	−0.735 **	−0.485 **	−0.147 **	−0.087	−0.019	−0.080	−0.674 **	−0.524 **
	*p*	<0.001	<0.001	0.004	0.092	0.708	0.193	<0.001	<0.001
Cosmetic appearance	R	−0.534 **	−0.300 **	−0.075	−0.046	0.017	0.001	−0.467 **	−0.416 **
	*p*	<0.001	<0.001	0.149	0.379	0.739	0.991	<0.001	<0.001
Extent of varicosity	R	−0.672 **	−0.384 **	−0.015	−0.113 *	0.024	−0.005	−0.546 **	−0.566 **
	*p*	<0.001	<0.001	0.777	0.029	0.638	0.937	<0.001	<0.001
Complications	R	−0.705 **	−0.507 **	−0.046	−0.126 *	0.008	−0.078	−0.605 **	−0.535 **
	*p*	<0.001	<0.001	0.374	0.014	0.873	0.208	<0.001	<0.001
AVVQ-H total score	R	−0.735 **	−0.450 **	−0.077	−0.120 *	−0.003	−0.074	−0.621 **	−0.591 **
	*p*	<0.001	<0.001	0.135	0.020	0.953	0.230	<0.001	<0.001

PF: Physical functioning, RP: Role limitations due to physical health, RE: Role limitations due to emotional problems, EF: Energy/fatigue, EWB: Emotional well-being, SF: Social functioning, PN: Pain, GH: General health. * *p* < 0.05, ** *p* < 0.01, R: correlation coefficient.

## Data Availability

Publicly available datasets were used in this study. These can be found in Figshare at https://doi.org/10.6084/m9.figshare.14865129.v1 (accessed on 10 January 2022).
